# Effects of Printing Paths on Compressive Strength of 3D-Printed Continuous Fiber-Reinforced Composite Lattice Unit Cell

**DOI:** 10.3390/polym17070850

**Published:** 2025-03-22

**Authors:** Min-Hyeok Jeon, Geun Sik Shin, Jun Yeon Hwang, Thuan Ho-Nguyen-Tan, Minkook Kim, Soon Ho Yoon

**Affiliations:** 1 Launch Vehicle Structures Team, Korea Aerospace Research Institute, 169-84 Gwahak-ro, Yuseong-gu, Daejeon 34133, South Chungcheong, Republic of Korea; mhjeon@kari.re.kr (M.-H.J.); 2 Institute of Advanced Composite Materials, Korea Institute of Science and Technology (KIST), 92 Chudong-ro, Bongdong-eup, Wanju-gun 55324, Jeonbuk, Republic of Korea

**Keywords:** 3D printing, printing path, continuous fiber, composite lattice, compressive strength

## Abstract

Three-dimensional printing is a highly promising manufacturing technology that enables easy production of complex shapes. Composite lattice structures are highly efficient, having the advantages of fiber-reinforced composites and the excellent structural performance of lattice configurations. Highly efficient structures can be developed by combining the benefits of 3D printing and composite lattice structures. This study examined the effect of printing path and axial angle in joint areas on the compressive strength of composite lattice unit cells fabricated via continuous fiber 3D printing. Compression tests were conducted to analyze deformation, failure modes, and causes of failure. A finite element model was used to analyze buckling behavior and establish design criteria. Results showed that the printing path significantly influenced failure mode and strength, while a fabrication method without a defect at the joint was important for improving structural performance. Finally, design criteria, in terms of the knockdown factor and in-plane bifurcation buckling behavior, were developed based on experimental and numerical results.

## 1. Introduction

Additive manufacturing (3D printing) is a promising technology that improves the design flexibility of complex geometries, which can be challenging to fabricate via traditional manufacturing processes. Users can customize structures with the required minimum volume, minimize material waste, and simplify the production process. Many studies have been conducted on using 3D printing to fabricate reinforcement structures to enhance the load-bearing capacity and shock absorption of structures subjected to compressive loads. A significant advantage of this approach is the ability to freely select and fabricate the size, thickness, and dimensions of internal reinforcement cells. Li et al. [1] used truss, honeycomb, chiral, and re-entrant cells for 3D-printed cellular lattice structures and conducted compression tests. The stiffness, strength, and energy absorption of the structures were compared, and design guidelines were provided for using 3D-printed reinforcement structures for compressive energy absorption. Zhou et al. [2] performed compression tests on structures with re-entrant, chiral, and double-arrowhead-shaped cells and demonstrated that the double-arrowhead shaped cells had superior compressive energy absorption performance compared with the other cells. Three-dimensional re-entrant honeycomb structures were also proposed to further enhance structural design efficiency [3]. He et al. [4] evaluated the compressive performance of re-entrant structures made using thermoplastics and short-fiber-reinforced materials, including PA, PA/CF, PEEK, and PEEK/CF. Zhang et al. [5] suggested a design methodology using compression tests, finite element analysis (FEA), and theoretical models for self-supporting 3D lattice structures aimed at energy absorption. Multi-material printing [1,6] and lattices filled with polyurethane foam [7] were also proposed to improve compressive properties.

In various fields, 3D-printing technologies have been used to fabricate reinforcement structures using thermoplastics and metal powders. Specifically, continuous fiber printing technology is rapidly developing. Continuous fibers are introduced into 3D printing to improve structural performance [8]. Three-dimensional printing with continuous carbon fiber combines the benefits of three-dimensional printing with the outstanding mechanical properties of carbon fiber. This process involves embedding continuous fiber tows within a thermoplastic matrix. Based on these advantages, many researchers have attempted to improve the mechanical and interlaminar strength of structures [9,10,11,12,13,14,15,16,17]. Furthermore, printing path planning and topology optimization have been conducted to improve the performance of load-bearing structures [18,19,20,21]. In particular, honeycomb and lattice-stiffened structures with continuous fibers have been studied [22,23]. For performance improvement and weight reduction, UAV wing structures and main bodies have been developed using continuous fiber 3D printing [24,25]. The internal structures were designed in a lattice form to improve the stiffness and reduce the weight. The lattice structure, made using composite materials, is highly efficient. It could withstand external loads through the high stiffness and strength of unidirectional fibers [26,27]. With these advantages, composite lattice structures have been used in various aerospace applications, such as satellite components, inter-stages, and long beams, to reinforce thin shells and skins [28,29,30]. These structures are usually manufactured through filament winding, but braiding and automated fiber placement have also been employed to fabricate various lattice configurations [31]. Thus, fabricating composite lattice structures, which have excellent properties, via 3D printing, which offers high design and manufacturing flexibility, enables the development and optimization of a wide range of lattice structures.

Zeng et al. [32] fabricated a honeycomb structure using continuous fiber printing and examined its in-plane and out-of-plane compressive behavior. A printing path without the fiber crossing was designed, and the compressive performance was investigated with the various dimensional parameters of the honeycomb cell. Dong et al. [33] fabricated continuous fiber-reinforced composite structures with square lattice (rhombus) and auxetic lattice forms and compared their compressive performance. Sugiyama et al. [34] examined the in-plane bending strength of various lattice structures. Cheng et al. [35] investigated the effect of fiber crossing in honeycomb composite structures on their bending performance. Wang et al. [36] developed orthogonal lattice structures with continuous fibers and compared their tensile properties based on the difference in the crossing of filaments. In addition, Wang et al. [37] reinforced flat panels with lattice structures and observed the effect of different printing paths on the bending strength and failure behavior. Ren et al. [38] optimized a grid-stiffened composite plate for buckling using the measured tensile mechanical properties of a lattice unit cell. Ma et al. [39] improved the mechanical performance of 3D-printed honeycomb and octet lattice structures made of recycled PLA by optimizing the printing parameters based on the stress state.

Many types of lattice structures made of 3D-printed continuous fiber composites have been researched. Although the printing path in the lattice joint area can significantly affect structural performances, recent studies have only focused on bending and tensile characteristics. However, lattice structures are most efficient under compressive loads. Furthermore, the mechanical properties of lattice unit cells should be measured and established as design criteria in advance to inform the overall structure design, but many researchers have skipped this step and proceeded directly to structural design and testing. Therefore, the effect of the axial angles of a lattice and the printing paths in its joint areas on the compressive strength of lattice unit structures should be studied.

In this study, a continuous fiber-reinforced composite lattice unit cell was fabricated to examine the effect of the axial angles of the lattice and the printing paths in its joint areas on their compressive strength. PLA-coated carbon fiber filaments and a 3D printer capable of coordinate customization were used. Specimens with three different printing paths were prepared considering the filament crossing in the joint area. Compression tests were performed on each specimen to identify differences in compressive strength due to variations in the printing path and axial angle. Subsequently, the buckling and failure behavior of the test specimens were examined. An equivalent finite element model was proposed for buckling behavior analysis. Two types of design criteria were presented using the model and the test results. First, the linear buckling load of each specimen was calculated using the proposed model, and a buckling knockdown factor was presented. Second, the in-plane bifurcation buckling behavior was analyzed using the finite element model. Based on the experimental and numerical results, the bifurcation point, which can be used in the design phase, was presented.

## 2. Compression Test

### 2.1. Specimen Preparation

**Figure 1:**
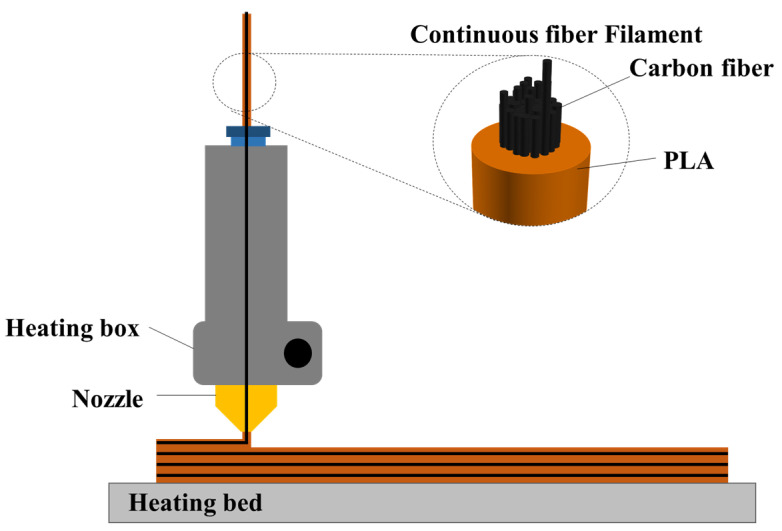
Three-dimensional printing of continuous fiber-reinforced composite.

A filament containing continuous fiber and PLA (Hanil Industrial Company, Namyangju, Republic of Korea) was printed using a 3D printer (Lincsolution Co., Anyang, Republic of Korea). The continuous carbon fiber was coated by PLA and the filament made up 20% of the weight. Figure 1 is the schematic diagram of the printing process. The filament, which consisted of T300 1K carbon fiber and thermoplastic PLA, was put through the nozzle. The nozzle temperature was set to 210 °C to melt the PLA, and the heating bed was set to 70 °C to stabilize the shape of the printed filament. The filament diameter was 1.2 mm, and the nozzle diameter was 1.5 mm. The filament was printed continuously, without cutting, and the nozzle moved along the designated path following G-code coordinates.

**Figure 2:**
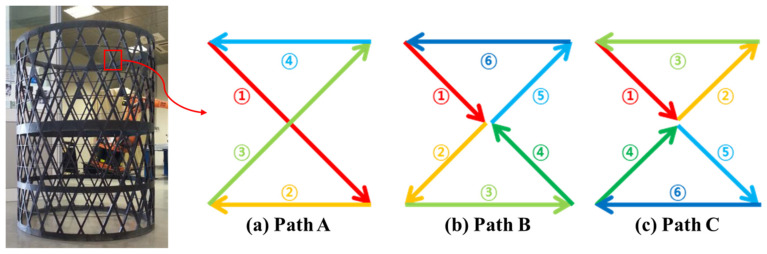
Composite lattice structure [21] and printing paths of unit cell (The number in the figure indicate printing sequence).

**Figure 3:**
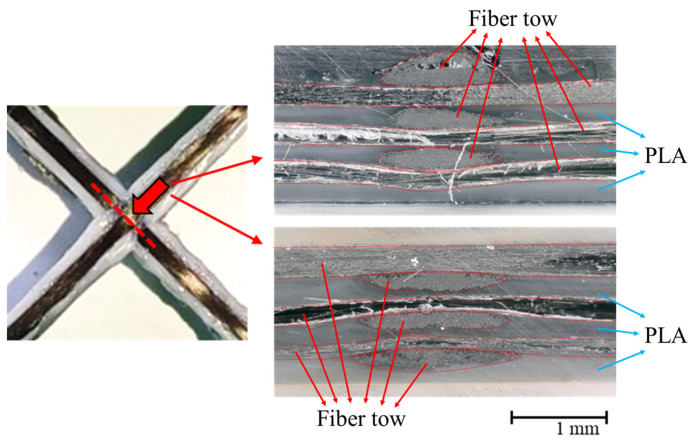
Cross-sections at joint of Path A specimen.

**Figure 4:**
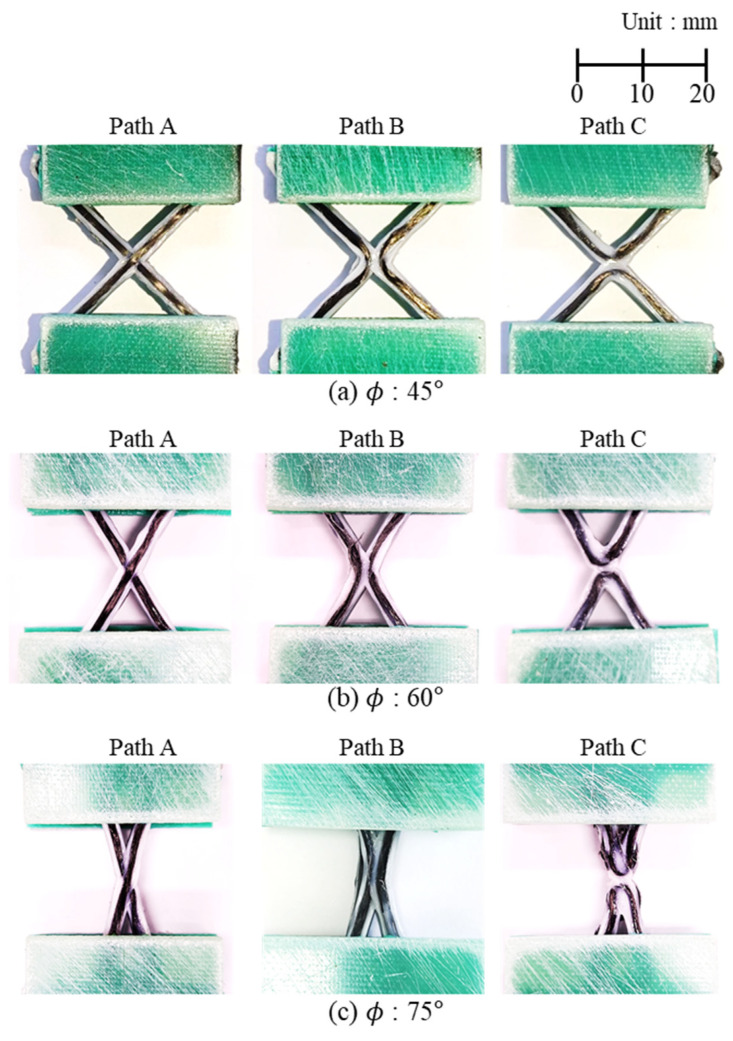
Specimens with different rib angles and printing paths.

**Figure 5:**
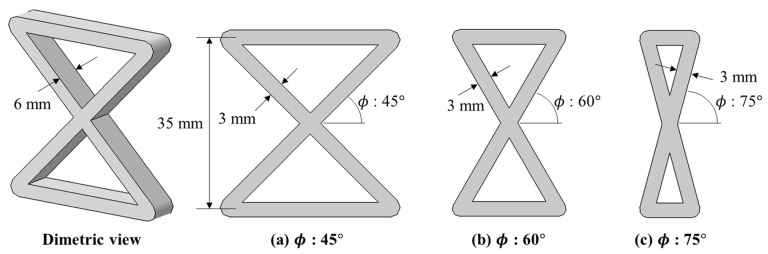
Specimen dimensions.

A composite lattice unit cell with continuous fibers has a joint area. This area can influence its capacity to withstand compressive load. Various printing methods can be used to design the joint area, with each affecting the load-bearing capacity differently [32,33,34,35,36,37,38,39]. The filament can cross at the joint area to form the unit cell, but it is also possible to design the printing path to avoid filament crossing. To address this, three different printing paths at the joints of the lattice unit cell were selected to examine the effect on compressive strength, as shown in Figure 2. Path A is a common truss or lattice structure where the filaments intersect at the joint. As depicted, the printing sequence 3 in Path A intersects with the already-printed sequence 1 within the same layer, leading to interference. The filament, printed in liquid form, can pass through the already-printed path before the PLA solidifies. The cross-sections of the Path A specimen were cut and polished to observe the intersection shapes using an optical microscope, as shown in Figure 3. The fibers bent, instead of remaining straight, at the intersections due to interference with the pre-printed filament. The thickness of both the intersection and non-intersection area was constant and the filament was printed regularly and stably. In Path A, the fibers bear axial compressive loads directly, but complex failure modes could occur due to the stress concentration and interference at the intersections [40,41]. Path B and C were designed to evaluate the compressive performance of joint configurations where the filament does not intersect and thus avoid interference at the joints. These paths allowed the fibers to carry loads without intersecting at the intersections. However, the printer nozzle moved only translationally (without rotation motion), and the fiber tows were twisted at the intersection points in the Path B and C specimens. Lattice structures under compressive loads are prone to buckling. Depending on the lattice and structural configuration, different types of buckling can occur, such as in-plane and global buckling. Additionally, the stiffness and buckling performance of a structure change according to the axial angles of the ribs that compose the structure [42,43,44]. Specimens with three different angles ϕ were fabricated to further examine the effects of three printing paths and three rib angles on axial stiffness and strength. Hence, a total of nine specimens were created, as shown in Figure 4. Figure 5 shows the specimen dimensions. The width and height of the 45° specimens were 35 mm each. For the specimens with other angles, the height was maintained at 35 mm; only the angle was modified. The specimens were fabricated with a sufficiently thick structure to ensure in-plane deformation under compressive loads and avoid out-of-plane deformation. Therefore, the width of the laminated ribs was 3 mm, and the overall thickness of each specimen was 6 mm.

### 2.2. Compression Test Procedure

**Figure 6:**
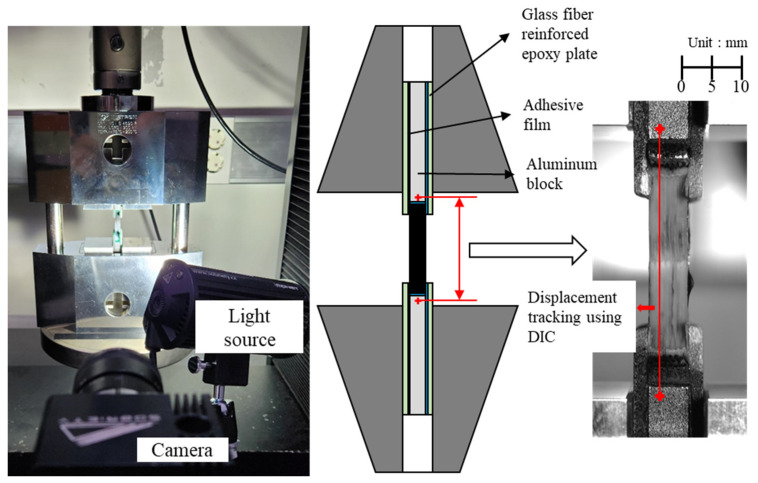
Compression test using ASTM D3410 test fixture and DIC.

A compression test was conducted with a cross-head speed of 2 mm/min using a 30 kN capacity universal testing machine, as shown in Figure 6, to measure the compressive strength of the fabricated specimens. With the use of the commonly applied flat compression plates for testing, the eccentric contact between the specimen and the fixture, along with misalignment, can result in nonuniform load distribution and unexpected fracture. An ASTM D3410 test fixture was used to apply stable and uniform compressive loads to the specimens [45]. This fixture transfers shear-induced compressive loads between a specimen and the tabs to the specimen. Glass fiber-reinforced epoxy tabs were attached to the specimens to fix the printed specimens in the test fixture securely, providing a sufficient surface area for uniform load transfer. Empty spaces were filled with aluminum blocks. The interfaces between the specimen, tabs, and blocks were bonded using an adhesive film. Although this testing method is highly stable for compressive load transfer, the total displacement measured from the crosshead of the testing machine includes the deformation of the specimen itself and additional displacement due to the slip at the specimen–fixture interface. Therefore, digital image correlation (DIC), which is a non-contact deformation measurement system, was used to track the displacement between the upper and lower aluminum blocks and thus accurately measure the compressive displacement of the specimen. During a compression test, a video was recorded to observe deformation and failure process of the specimen.

## 3. Finite Element Analysis

**Figure 7:**
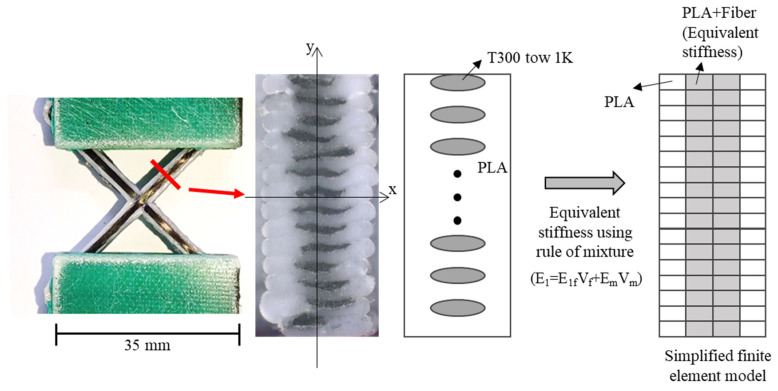
Simplified model using rule of mixture and equivalent stiffness.

**Figure 8:**
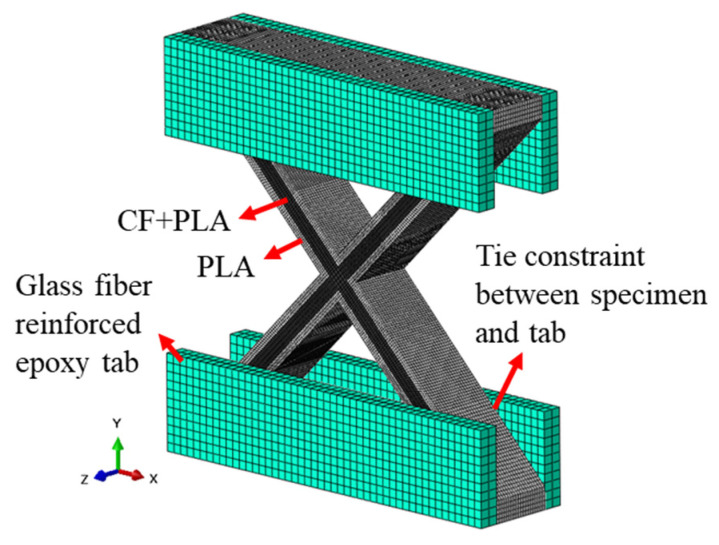
Modeling of specimens and section assignment.

**Figure 9:**
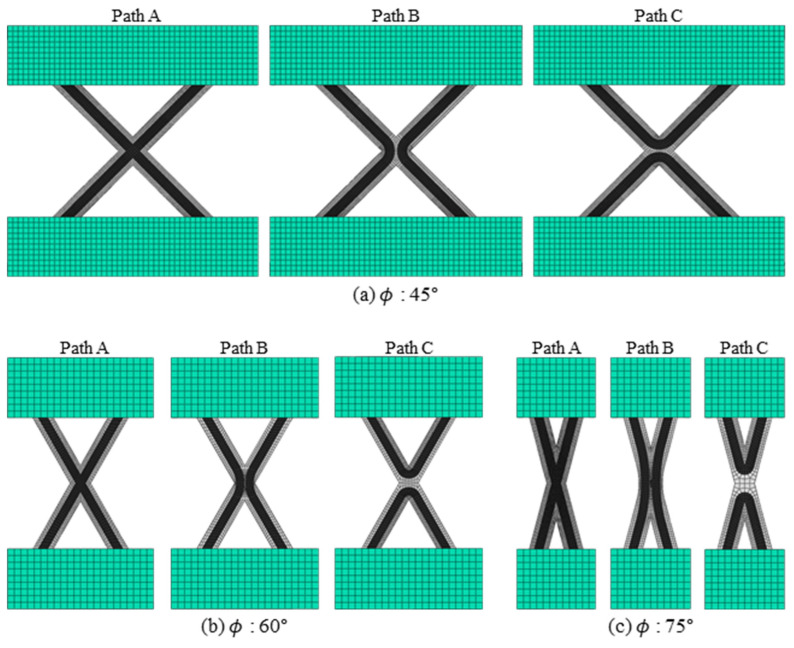
Finite element models.

Finite element analysis was performed to examine the failure modes of the lattice structure and to propose design criteria for the unit cell. Given that lattice specimens under compressive load are prone to buckling, this analysis specifically focused on investigating their buckling behavior. As shown in Figure 7, the actual shape of the PLA-coated carbon fiber filament, including the fiber and resin distribution, was observed using an optical microscope. The cross-section at the boundary of the PLA extruded from the nozzle and was nearly elliptical, with the fibers and the PLA matrix regularly located. The fibers were eccentrically positioned at the top of the filament. In the test specimens, the thickness was large relative to the width of the ribs. As observed in Figure 7, the out-of-plane stiffness (EI_xx_) is significantly lower than the in-plane stiffness (EI_yy_), suggesting that in-plane deformation would predominantly occur. Consequently, a simplified equivalent model was proposed to use computational resources efficiently and represent in-plane deformation adequately. Calculating the stress distribution in detail required a meso-scale model with real cross-sections of the fiber and resin. However, because this study focused on buckling analysis specifically in the in-plane domain, the cross-section was modeled as a rectangle despite the elliptical shape of the PLA. The elements corresponding to the central region of the specimen cross-section, which aligned with the width occupied by the fibers, were assigned equivalent stiffness values based on the fiber and PLA area and stiffness to represent the equivalent in-plane stiffness effectively. The equivalent stiffness was calculated using the stiffness of the T300 fibers and PLA, combined through the rule of mixtures. The stiffness of the T300 fibers, provided by the Toray technical data sheet [46], was 230 GPa, and the stiffness measured through the tensile testing of the PLA fabricated using the same printer was 1 GPa. As shown in Figure 8, the specimen and the glass fiber-reinforced tabs were modeled, and the adhesive film between the specimen and the tabs was replaced with a tie constraint. The models of the specimens with different angles and printing paths are shown in Figure 9.

## 4. Results and Discussion

### 4.1. Test Results

**Figure 10:**
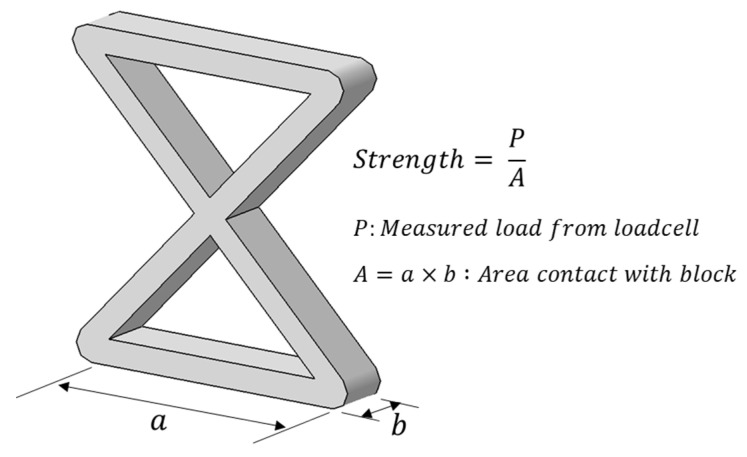
Calculation of strength.

**Figure 11:**
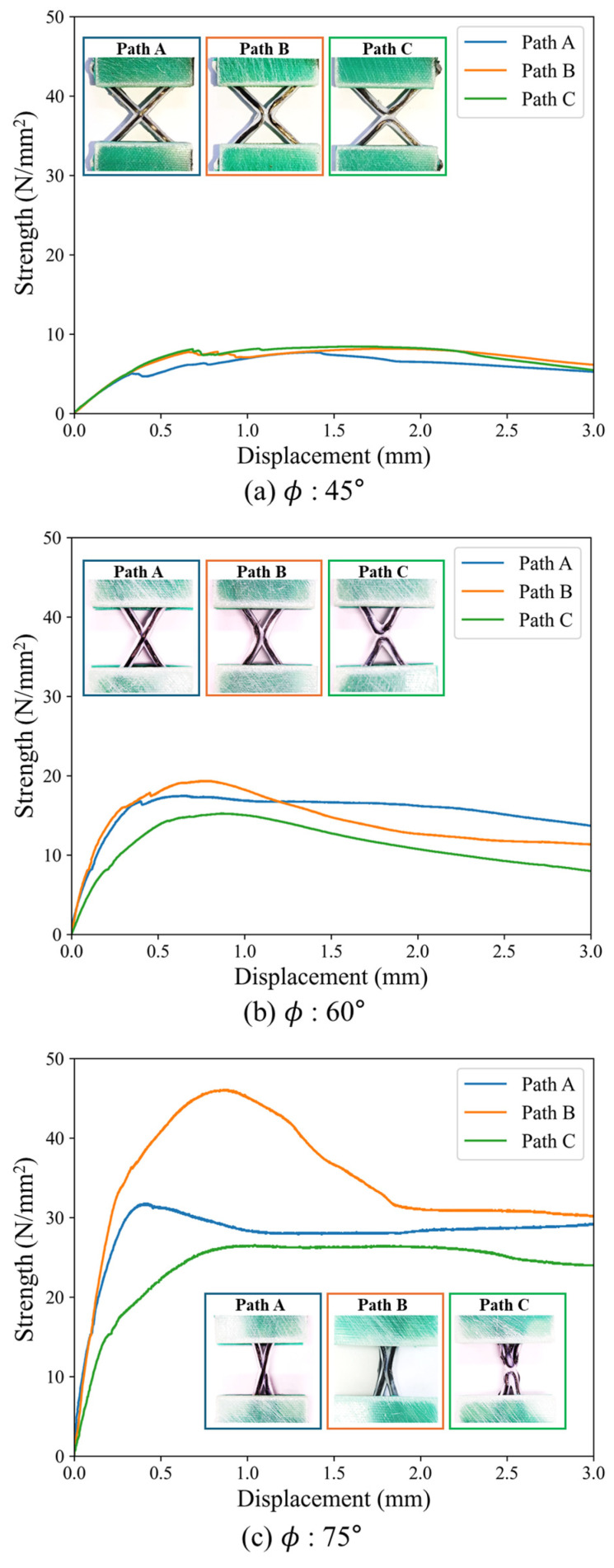
Strength to displacement curve from compression tests.

Compression tests were conducted on the specimens with different printing paths and axial angles, and their compressive strengths were compared. The load was divided by the cross-sectional area of the specimen in contact with the aluminum block to calculate and compare the strength data with respect to the specimen dimensions, as shown in Figure 10. Figure 11 shows the curve of strength against displacement.

Among the ϕ = 45° specimens, the Path C specimen had the highest compressive strength, although there was no significant variation observed among the different paths. As the angle increased, the axial stiffness of the specimens increased, leading to an overall increase in the initial stiffness and maximum compressive strength. Although Path C exhibited the highest strength at 45°, it showed the lowest strength at 60° and 75°, compared to other paths. As the angle increased, Path B showed higher strength compared with the specimens of the other patterns and its strength increased more significantly.

**Figure 12:**
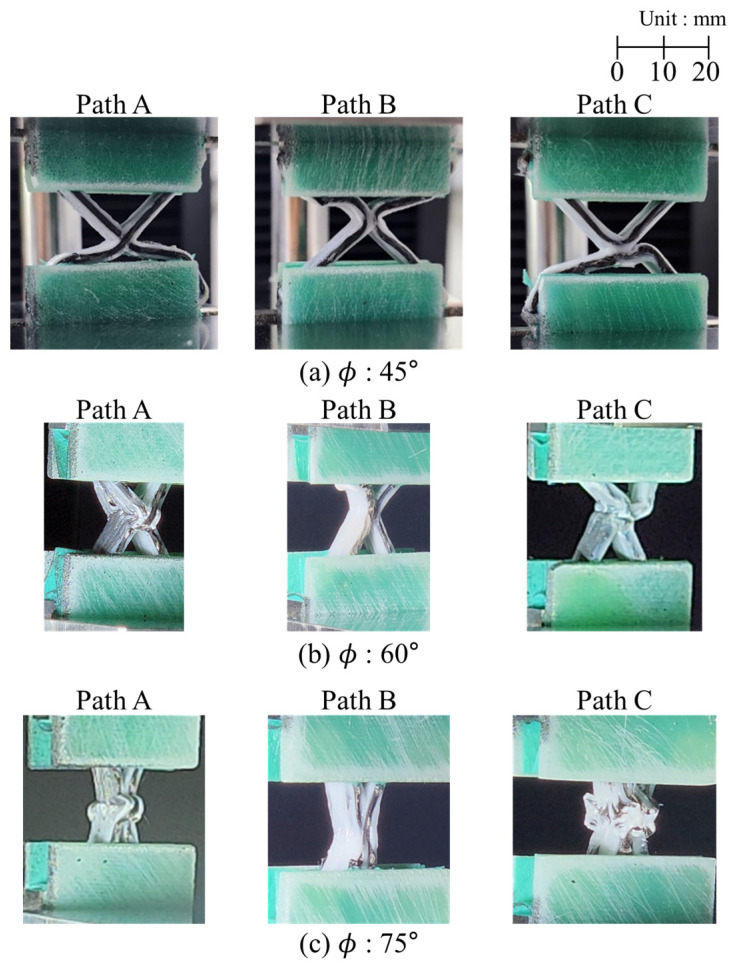
Deformation and failure modes.

Figure 12 shows the failure modes observed after reaching the maximum strength of the specimens. Lattice structures can experience in-plane local buckling of the ribs, out-of-plane buckling of the entire specimen, and fracture at the intersections. Given the sufficiently thick thickness of the specimens in this study, their out-of-plane buckling was not considered. If a joint has enough strength, then in-plane buckling is expected; however, if the joint cannot bear the load sufficiently, failure or fracture at the joint will occur before in-plane buckling. Regardless of printing path, only in-plane deformation was observed in the ϕ = 45° specimens. After in-plane buckling, the matrix exhibited plasticity, leading to a decrease in load after the load reached the maximum strength of the specimen. For the ϕ = 60° and ϕ = 75° specimens, only the Path B specimen primarily exhibited in-plane failure; from the recorded video, it was observed that the interlaminar fracture initiated first and led to entire failure, with plasticity of the PLA of the Path A and C specimen.

Table 1 summarizes and compares the maximum strengths and failure modes of specimens with axial angles and printing paths. Little variation in strength was seen between the ϕ = 45° specimens, where only in-plane buckling occurred. However, among the ϕ = 60° and ϕ = 75° specimens, the specimens exhibiting in-plane buckling showed higher strength, whereas those exhibiting interlaminar failure had lower strength. Therefore, because the Path B consistently exhibited the highest strength at all angles with in-plane buckling, it was considered the most efficient structure. As the angle increased, the axial stiffness increased and the rib length decreased, making the structure more resistant to in-plane deformation and increasing the in-plane buckling load. The Path B specimens were also the only ones able to sustain high in-plane buckling loads; the Path A and Path C specimens did not have sufficient joint strength at higher angles, which reduced the structural stability.

**Figure 13:**
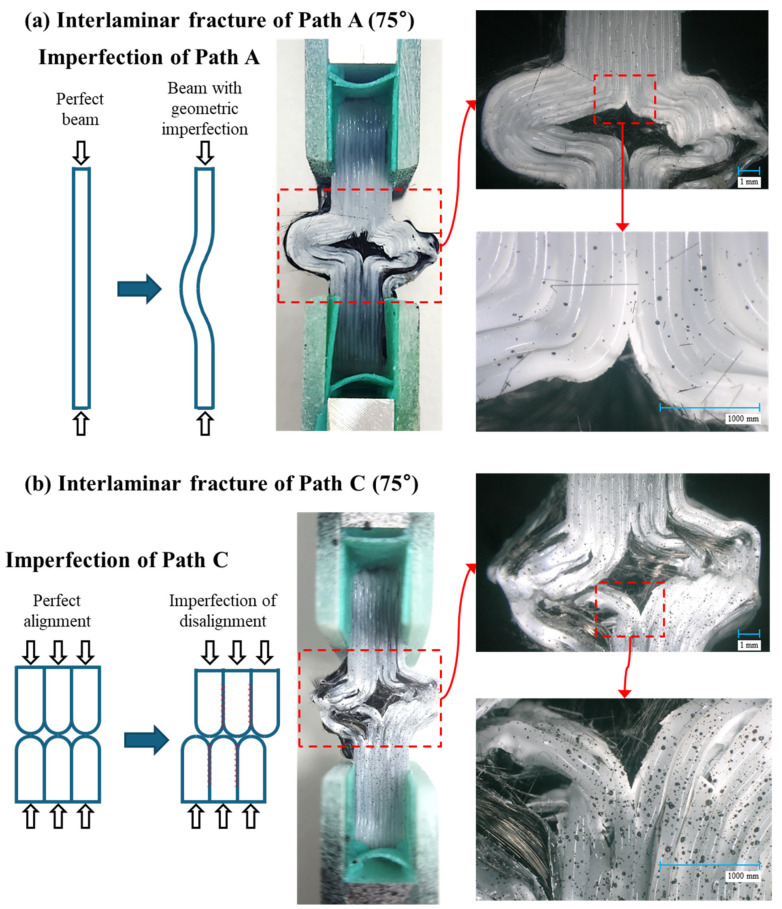
Interlaminar failure and possible fracture mechanisms in Path A and C.

The possible causes of the reduced joint strength in the Path A and Path C specimens are supposed in Figure 13. For the Path A, as shown in Figure 13a, the bending shape of the fibers at the intersections was observed. When the fibers are not straight but curved, these bends act as an initial geometric imperfection, which can degrade structural performance by contributing to reduce the buckling load or strength. In the case of the Path C, the filament was not linked at the joint between the upper and lower triangle cell, because it was not continuously printed at once, as illustrated in Figure 13b. If the upper and lower filaments are perfectly aligned, then the contact surface can uniformly bear and share the compressive load. However, in the presence of small misalignments, as shown in the figure, the load can create cracks between layers. As the angle increases, although the fibers can directly carry more axial load, the matrix at the joints ultimately bears the total load. Thus, the maximum load that a specimen can withstand is determined by the shape and properties of the matrix. Additionally, during printing, the nozzle moved translationally only without rotating, causing the fibers to twist at the corners. Consequently, with an increase in the angle, the fibers in the Path C joint will be highly twisted and curved. Furthermore, in the Path C specimen with axial angles of 60° and 75°, the fiber tow did not follow the designated printing path due to those defects. By contrast, for Path B, as the printing angle increased, the twisting and bending angle of the fiber tows in the filament joint area decreased and the fiber tows became closer to straight. In Path C, as the angle increased, the defects enlarged, leading to a reduction in strength. However, in Path B, the defects became smaller, resulting in greater structural robustness and strength.

### 4.2. Design Criterion Using Knockdown Factor

**Figure 14:**
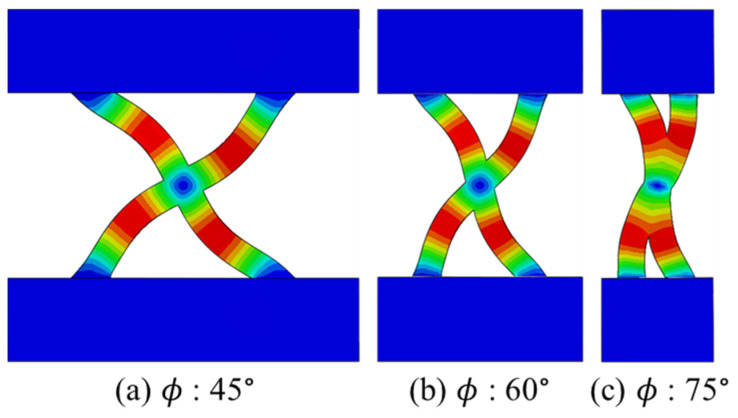
Linear buckling mode (The color indicate magnitude of eigenvector).

Linear buckling analysis was performed on the lattice specimens, and a knockdown factor was calculated to provide design guidelines. The knockdown factor (γ) is

$$Knockdown\;factor\;\left(\gamma \right)={\left(P_{cr}\right)}_{imperfect}/{\left(P_{cr}\right)}_{perfect}$$

where ${\left(P_{cr}\right)}_{perfect}$ is the linear buckling load of a perfect model without any defects, and ${\left(P_{cr}\right)}_{imperfect}$ is the actual buckling load measured from the real structures. A mesh convergence study was performed, and the finite element model was verified by comparing test results. The finite element model with a different angle followed the elastic behavior of the tested specimens well. Linear buckling analysis was conducted using the models proposed in Section 3 and the mode shapes shown in Figure 14. For the models with the same angles, the same mode shape was observed regardless of the printing path. Therefore, only the shape for Path A is presented here as a representative example for each angle.

**Figure 15:**
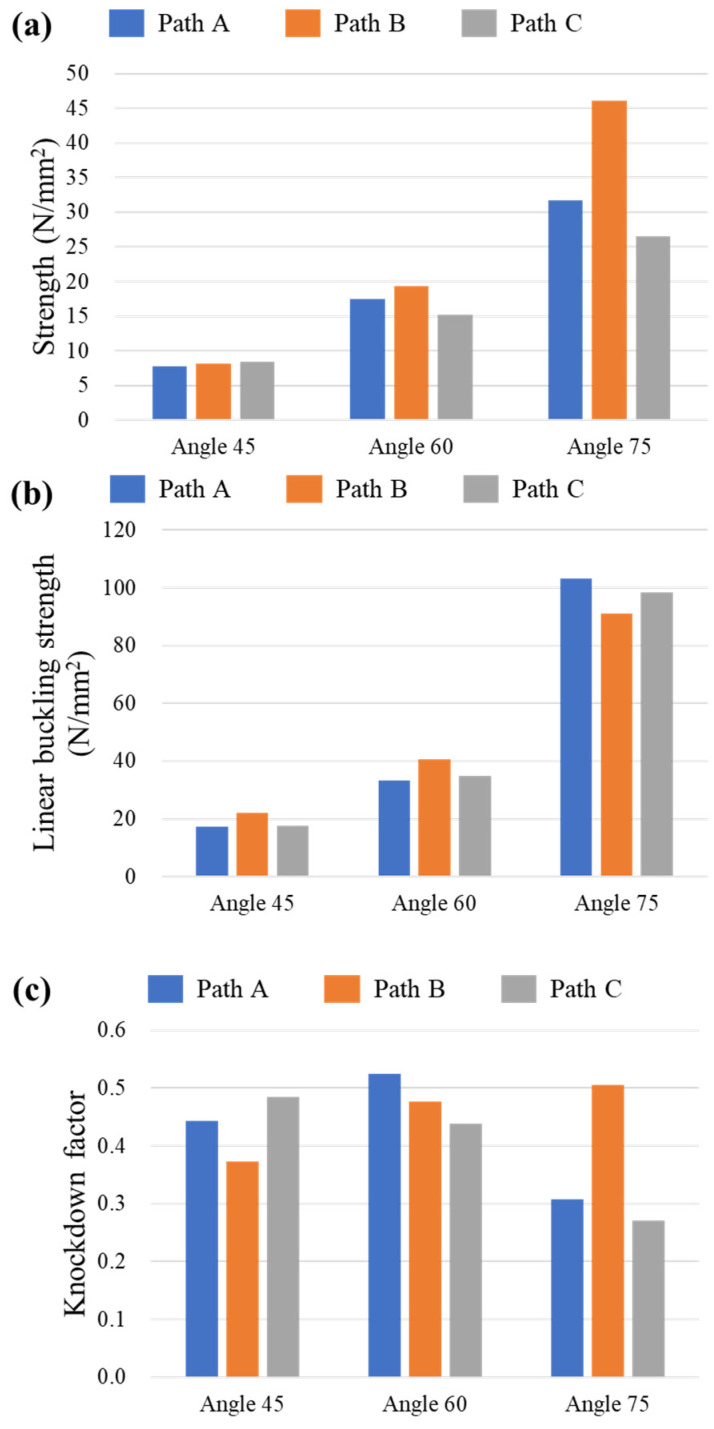
(**a**) Maximum strength, (**b**) linear buckling strength, and (**c**) knockdown factor.

With a rib width of 3 mm and a thickness of 6 mm, the specimens had a sufficient thickness relative to their rib width. Because they were more susceptible to in-plane deformation than out-of-plane deformation, the first buckling mode was in-plane buckling. Figure 15 presents the strength, linear buckling strength, and knockdown factor of the specimens. Linear buckling load was expressed by linear buckling strength to compare with the strength of the specimen. Figure 15a shows the maximum strength measured during the compression test. Figure 15b shows the linear buckling strength. Among the ϕ = 45° specimens, the Path B specimen had the highest calculated buckling load, whereas the path A specimen exhibited the highest buckling load. However, the actual test results showed that the structural performance of Path B improved significantly with an increase in angle. In Figure 15c, the knockdown factor for Path A and Path C decreased with an increase in angles, whereas for Path B, the knockdown factor increased with the angle. This trend aligned with the test results in Section 4.1, where interlaminar failure was observed in the Path A and Path C specimens with a ϕ of 60° and 75°, leading to lower strengths compared with those of the Path B specimens. The interlaminar failure reduced the structural performance of these paths. As suggested by the knockdown factor, although the buckling load could increase, the joints did not support sufficient loads up to the point of in-plane buckling, resulting in worse design criteria. Design criteria were developed for 3D-printed composite lattice structures under axial compressive loads based on the linear buckling analysis results and actual strengths.

### 4.3. Bifurcation Buckling Analysis for Path B

The knockdown factor presented in 4.2 can be used in the design of a structure with a lattice unit cell. An engineer can choose a printing path that satisfies the design requirement. However, the Path B specimens had superior compressive properties relative to the Path A and C specimens. The dimensions did not differ between specimens; only the printing path varied. Additionally, the printing path can be customized easily. Path B only showed in-plane deformation, regardless of angle, and the best structural performance. This was considered as failure due to bifurcation buckling in this paper. Therefore, the in-plane bifurcation buckling behavior for Path B was examined to propose additional design guidelines in detail.

In beams under a pure compressive load, the strain along the sides increases uniformly until the bifurcation buckling point. When a compressive load is applied to a lattice specimen, in addition to the pure compressive load, moments are generated due to the distance between the load and the support points. This moment causes an initial difference in the axial strain along the sides of the ribs from the beginning.

**Figure 16:**
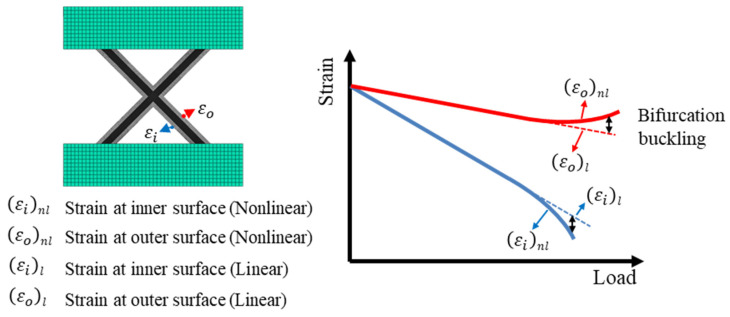
Definition of bifurcation buckling for lattice unit cell.

In this study, finite element analysis (FEA) was conducted on the lattice structure under a compressive load to define its bifurcation buckling point. Both linear and nonlinear (considering geometric nonlinearity) analyses were performed to compare strains. As shown by the linear static analysis results in Figure 16, the strain increased linearly with the load. At low loads, the linear and nonlinear analyses showed similar strains. However, in the nonlinear analysis, as the load increased, the bending deformation due to the moment was more significantly accounted for, leading to a bifurcation point where the strain increased or decreased rapidly. The strain difference between the linear and nonlinear analyses was considered the criterion for defining this bifurcation point, as graphically depicted in Figure 16.

**Figure 17:**
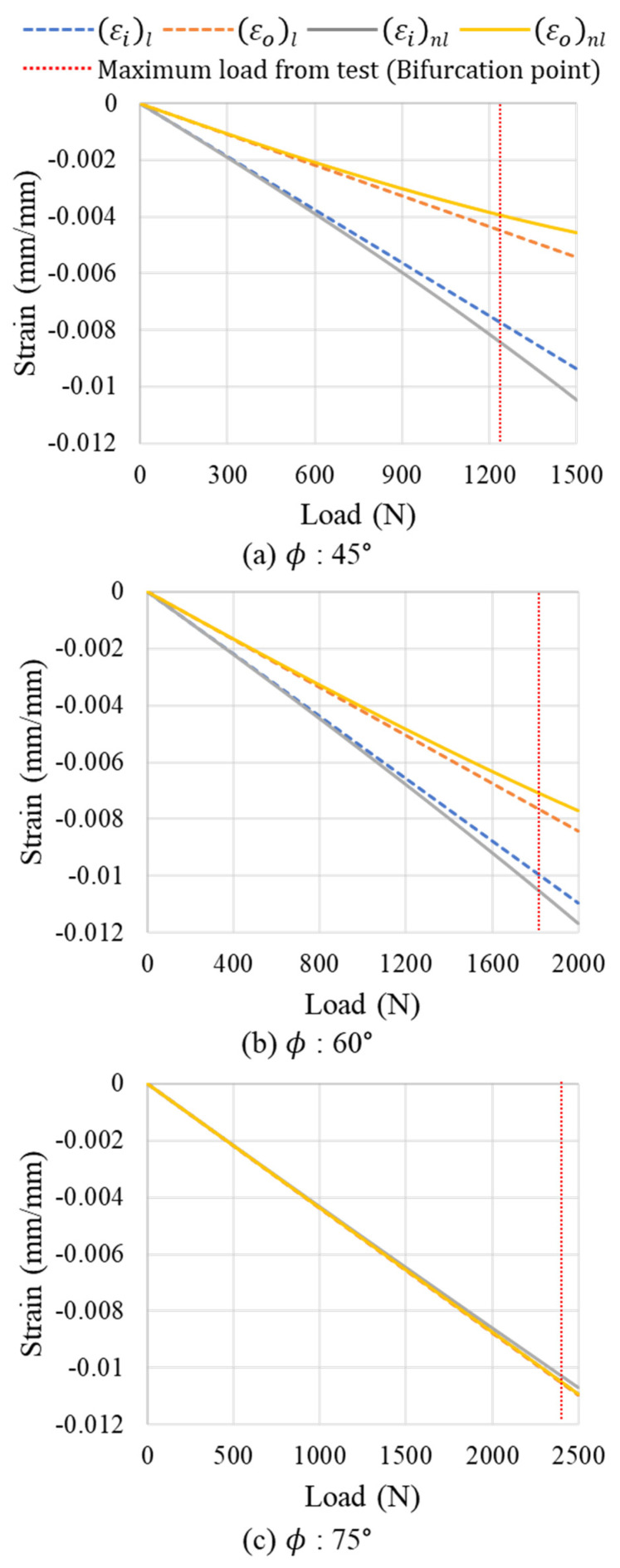
Strain–load relationship for bifurcation buckling point definition.

The axial strain in the ribs of the Path B specimens was calculated under compressive loads at different angles. The strain load relationships for these models are shown in Figure 17. As mentioned in the preceding discussion, the strain values from the linear and nonlinear analysis were similar at low loads. However, as the load increased, the difference between the linear and nonlinear analysis strains increased after the bifurcation point.

With an increase in angle, the axial stiffness increased and the distance between the load and support points decreased, which reduced the moment and bending deformation. When bending deformation occurs in addition to compression, the stress and deformation at specific points increase, particularly in the side under compressive stress, leading to reductions in structural stiffness and strength. As shown in Figure 17a, for the ϕ = 45° model, the difference between the linear and nonlinear strains increased with the load after the bifurcation point. In the ϕ = 60° model, this difference decreased, and at ϕ = 75°, the difference was not considerable. Therefore, as the angle increased, the bending deformation decreased, leading to a decrease in the difference in the strain distribution. Consequently, the structural stiffness and strength improved, and failure occurred primarily due to in-plane deformation and buckling under compression.

The bifurcation point is defined as the difference in strain between the linear and nonlinear analyses at the maximum load measured during the compression test. These differences are summarized in Table 2. As the angle increased, the maximum strength increased and the strain difference at bifurcation point decreased. At lower angles, both compression and bending deformations were present, whereas compressive deformation become dominant at higher angles. This resulted in bifurcation buckling occurring at higher loads and an overall increase in strength.

## 5. Conclusions

In this study, compression tests were performed on 3D-printed continuous fiber-reinforced composite lattice structures, and the effects of the printing paths and angles on their compressive strength were examined. The specimens were fabricated using PLA filaments with carbon fibers and tested using a stable compression test fixture.

The compression test results showed that the specimens with nonintersecting fiber paths, specifically Path B, exhibited the highest strength. The relationship between the strength and failure modes indicated that interlaminar fracture reduced structural strength, whereas in-plane deformation and buckling enhanced strength. Manufacturing defects in the joint areas induced interlaminar fracture and reduced the strength of the specimens.

The buckling knockdown factor was presented by comparing the measured actual strength of the specimens with the linear buckling analysis results. Although the buckling loads for the Path A and Path C specimens were lower than those for Path B at a small angle, their buckling loads exceeded those of Path B as the angle increased. However, the defects at their joints reduced their actual strengths. The knockdown factors of the Path A and C specimens also decreased as the angle increased. Finally, Path B was found to be the best printing path for the joint of the 3D-printed lattice structures under compressive loads.

Bifurcation buckling behavior was examined for the Path B specimens, which failed due to in-plane buckling. As the angle increased, the distance between the loading point and the support point decreased. Consequently, bending and bifurcation deformation decreased, eventually improving structural performance.

Two types of guidelines for failure prediction were introduced in this study. The knockdown factor derived from the experimental and numerical procedures can be used in the design phase. Only the linear buckling load calculated using the proposed finite element model is required to simply design a structure under compression. Furthermore, when structures composed of repetitive lattice unit cells are deformed by external loads, the deformation can be complex and uneven. In this case, the bifurcation buckling point can be used to predict the failure of the arbitrary region and the structural strength.

The 3D-printed composite lattice structures achieved the best structural performance when the filaments were arranged to avoid crossing. Defects in the joint area significantly reduced strength. Although this study provides knockdown factors as design criteria for 3D-printed composite lattice structures, further research is required to establish design criteria for variations in grid size, width-to-thickness ratio, and other geometric parameters.

## Figures and Tables

**Table 1:** Comparison of strength and failure mode.

		Strength (N/mm^2^)	Failure Mode
***ϕ*** = 45°	Path A	7.7	In-plane buckling
	Path B	8.2	In-plane buckling
	Path C	8.5	In-plane buckling
***ϕ*** = 60°	Path A	17.5	Interlaminar fracture
	Path B	19.3	In-plane buckling
	Path C	15.3	Interlaminar fracture
***ϕ*** = 75°	Path A	31.8	Interlaminar fracture
	Path B	46.1	In-plane buckling
	Path C	26.6	Interlaminar fracture

**Table 2:** Bifurcation buckling point for Path B specimens.

	ϕ = 45°	ϕ = 60°	ϕ = 75°
Inner surface	9.0%	5.4%	1.8%
Outer surface	12.4%	7.1%	0.2%

## Data Availability

The data that support this study can be available upon reasonable request.
